# The crystal structure of the endoglucanase Cel10, a family 8 glycosyl hydrolase from *Klebsiella pneumoniae*

**DOI:** 10.1107/S2053230X16017891

**Published:** 2016-11-25

**Authors:** Ayman Attigani, Lifang Sun, Qing Wang, Yadan Liu, Dingping Bai, Shengping Li, Xiaohong Huang

**Affiliations:** a Key Laboratory for Integrated Chinese Traditional and Western Veterinary Medicine and Animal Healthcare, College of Animal Science, Fujian Agriculture and Forestry University, 15 Shang Xia Dian Road, Fuzhou 350002, People’s Republic of China; b State Key Laboratory of Structural Chemistry, Fujian Institute of Research on the Structure of Matter, Chinese Academy of Sciences, 155 West Yangqiao Road, Fuzhou 350002, People’s Republic of China

**Keywords:** cellulases, *Klebsiella pneumoniae*, carboxymethyl cellulase, cellulose biosynthesis, crystal structure, Cel10

## Abstract

Cellulases are produced by microorganisms that grow on cellulose biomass. Here, a cellulase, Cel10, was identified in a strain of *Klebsiella pneumoniae* isolated from Chinese bamboo rat gut. Analysis of substrate specificity showed that Cel10 is able to hydrolyze amorphous carboxymethyl cellulose (CMC) and crystalline forms of cellulose (Avicel and xylan) but is unable to hydrolyze *p*-nitrophenol β-d-glucopyranoside (*p*-NPG), proving that Cel10 is an endo­glucanase. A phylogenetic tree analysis indicates that Cel10 belongs to the glycoside hydrolase 8 (GH8) subfamily. In order to further understanding of its substrate specificity, the structure of Cel10 was solved by molecular replacement and refined to 1.76 Å resolution. The overall fold is distinct from those of most other enzymes belonging to the GH8 subfamily. Although it forms the typical (α/α)_6_-barrel motif fold, like *Acetobacterxylinum* CMCax, one helix is missing. Structural comparisons with *Clostridium thermocellum* CelA (CtCelA), the best characterized GH8 endoglucanase, revealed that sugar-recognition subsite −3 is completely missing in Cel10. The absence of this subsite correlates to a more open substrate-binding cleft on the cellooligosaccharide reducing-end side.

## Abstract 2

A cellulase from *K. pneumoniae* was cloned, expressed, purified and shown to be an endoglucanase. Its structure was determined at 1.76 Å resolution and comparisons with homologous enzymes provide information on substrate specificity.

## 1. Introduction   

Cellulose is the most abundant organic compound on earth and is the major polysaccharide component of plant cell walls. Cellulose fibres comprise crystalline and amorphous arrays of polysaccharide chains. Effective hydrolysis of cellulose requires three types of cellulases, namely endo-(1,4)-β-d-glucanase (EC 3.2.1.4; carboxymethylcellulase or CMCase), exo-(1,4)-β-d-glucanase (EC 3.2.1.91; cellobiohydrolase, avicelase, microcrystalline cellulase or β-exoglucanase) and β-glucosidase (EC 3.2.1.21), which must act synergistically to achieve the degradation of crystalline cellulose (Tomme *et al.*, 1995 ▸). Although a large number of microorganisms are capable of degrading cellulose, only a few of them produce significant quantities of cell-free bioactive compounds capable of completely hydrolyzing crystalline cellulose *in vitro* (Bai *et al.*, 2012 ▸). Numerous studies have reported the degradation of cellulosic materials, but only a few have examined which microorganisms might offer economical benefits (Yamada *et al.*, 2011 ▸). Microbes play a vital role in the degradation of cellulose and some animals have achieved effective cellulose utilization by developing symbiotic relationships with microbes that are present in their gut as the primary cellulolytic agent (Watanabe & Tokuda, 2001 ▸).

The Chinese bamboo rat (*Rhizomyssinensis*) is well known for its dietary oddities: it is a bamboo specialist within the mammalian order Herbivores that possesses a gastrointestinal tract typical of carnivores. It consumes the roots and shoots of bamboo and other highly fibrous plants each day (Musser & Carleton, 2005 ▸; Anderson & Jones, 1984 ▸; Clarke, 2010 ▸). By sequence analysis of the conserved 16S rRNA, a molecular marker for the identification of bacterial species (Srinivasan *et al.*, 2015 ▸), a bacterium isolated from the gastrointestinal tract of the Chinese bamboo rat was identified as a *Klebsiella* strain and named *Klebsiella* 10. A cellulase gene was cloned from the *Klebsiella* 10 chromosomal DNA using a pair of special primers and was thus named Cel10. The gene encodes a protein of 310 amino-acid residues, including a signal-peptide segment (residues 1–23), and has a mature molecular weight of 35 kDa. The protein is predicted to be a member of glycoside hydrolase family 8 (GH8) according to the CAZy database (http://www.cazy.org). Based on the facts that genes encoding cellulases are essential in bacteria and that the proteins are putative targets for enzyme development, there have been numerous studies of the three-dimensional structures of cellulases (Dominguez *et al.*, 1995 ▸; Ducros *et al.*, 1995 ▸; Clarke, 2010 ▸). Previous analyses have provided a basis for modelling homologous GH8 cellulases and the architecture of the active-site cleft, which presents at least five glucosyl binding subsites and explains why GH8 cellulases cleave oligosaccharide polymers that are at least five d-glucosyl subunits in length. Furthermore, the structure of CtCelA (CelA) allows comparison with (α/α)_6_-barrel glycosidases that are not related in sequence, suggesting a possible, albeit distant, evolutionary relationship between different families of glycosyl hydrolases (Alzari *et al.*, 1996 ▸).

Cellulases, by virtue of their ability to degrade cellulose substantially, are key industrial enzymes of the 21st century. There is a considerable drive to uncover new enzymes, to determine their three-dimensional structures and assess them for cellulose deconstruction. Thus, recombinant Cel10 was studied in order to understand its structure–function relationship with respect to cellulolytic activity. Here, we present the cloning, expression, purification, crystallization and X-ray diffraction analysis of a cellulase from the cellulolytic bacterium *K. pneumoniae* found in the gut of the Chinese bamboo rat.

## 2. Materials and methods   

### 2.1. Cloning and expression of Cel10   

The DNA encoding amino acids 24–333 of Cel10 was amplified by polymerase chain reaction (PCR) using *K. pneumoniae* genomic DNA as a template and the gene-specific forward primer CLE-BamH1 (5′-CGGGAT­CCGATACGGCCTGGGAGCGCTA-3′) and reverse primer CLE-XhoI (5′-CCGCTCGAGCTAACGCTGATCCTGTTTCG-3′) (Table 1 ▸). The PCR product was cloned into the expression vector pET-32a [modified by inserting a *Tobacco etch virus* (TEV) protease cleavage site inside the NcoI site] with BamHI and XhoI. *Escherichia coli* strain DH5α (Novagen) was used for plasmid amplification, which was confirmed by DNA sequencing. The recombinant plasmid was then transformed into *E. coli* strain BL21 (DE3) (Novagen) for protein expression. Cells were grown in Luria–Bertani (LB) medium plus 100 mg l^−1^ ampicillin with shaking at 310 K for 6 h, and expression of Cel10 was induced by adding isopropyl β-d-1-thiogalactopyranoside to a final concentration of 0.3 m*M* when the cells reached the mid-log phase of growth (optical density at 600 nm of 0.6–0.8); the cells were then grown overnight with shaking at 289 K.

### 2.2. Protein production and purification   

**Figure 1:**
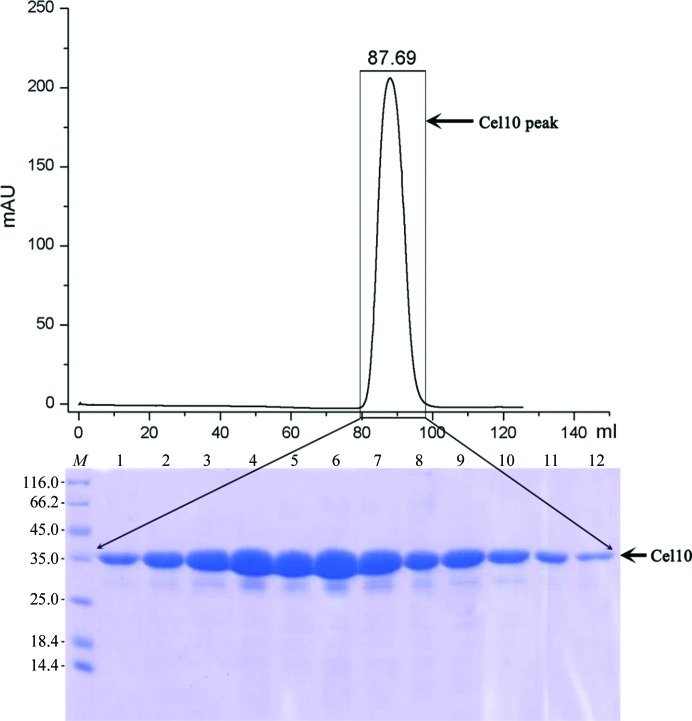
SDS–PAGE analysis of purified recombinant Cel10. Top: size-exclusion chromatography (SEC) chromatogram of Cel10 from the final purification column showing a notable peak. Bottom: SDS–PAGE gel of the peak fraction. The protein fractions were resolved on a gradient SDS–PAGE gel (15%) and stained using Coomassie Blue for visualization. Lane *M* contains molecular-weight markers (labelled in kDa).

Cel10 protein was purified using a four-step protocol: an Ni^2+^-affinity chromatography step, cleavage of the N-terminal 6His-Trx tag with TEV protease, removal of the cleaved tag by a second Ni^2+^-affinity chromatography step and finally size-exclusion chromatography (SEC), which was performed on an ÄKTApurifier (GE Healthcare) using SEC programmes according to previously described procedures (Bryan *et al.*, 2011 ▸). The cells containing expressed Cel10 were harvested by centrifugation at 7000*g* for 5 min at 277 K. The cell pellets were thawed on ice, resuspended in lysis buffer consisting of 50 m*M* MES pH 6.0, 500 m*M* NaCl, 5% glycerol supplemented with 5% Tween 20 and 0.1 µm PMSF, and disrupted by ultrasonication on ice for 30 min. Cell debris was removed by centrifugation at 20 000*g* for 30 min at 277 K using a Beckman Avanti J-301 centrifuge. The resulting supernatant was loaded onto nickel Sepharose affinity resin. After the flowthrough had been discarded, the column was washed with lysis buffer containing a linear gradient from 20 to 100 m*M* imidazole, and target proteins were eluted from the column using lysis buffer plus 500 m*M* imidazole. 6His-TEV protease was added to the eluted protein at a ratio of 1:10(*w*:*w*) to cleave the 6His-Trx tag. The 6His-TEV protease and 6His-Trx tag were then removed by a second Ni^2+^-affinity chromatography step. The resulting protein was further purified by SEC (Superdex 200, GE Healthcare) using a buffer consisting of 50 m*M* MES, 100 m*M* NaCl pH 6.0, 5% glycerol. The SEC chromatogram showed one peak at 87.69 ml consistent with the molecular weight of Cel10 (35 kDa). After SDS–PAGE analysis (Fig. 1 ▸), the purified Cel10 was concentrated for crystallization to 28 mg ml^−1^ using an ultrafiltration system (Millipore, 30 kDa cutoff). The protein concentration was determined by the Bradford method using bovine serum albumin (BSA) as the standard (Bradford, 1976 ▸).

### 2.3. Substrate specificity   

The Cel10 activity was determined according to a previously described method (Saratale *et al.*, 2010 ▸, 2012 ▸). Endoglucanase activity was determined using a reaction mixture consisting of 1 ml enzyme solution (4 mg ml^−1^) with 2 ml 1%(*w*/*v*) CMC in McIlvaine’s buffer (0.1 *M* citric acid/0.2 *M* phosphate buffer pH 5) and incubated at 323 K for 30 min followed by the addition of 1.5 ml dinitrosalicylic acid reagent. Cellulolytic activities towards Avicel for avicelase activity and towards xylan for xylanase activity were measured by replacing the CMC from the earlier assay with 1%(*w*/*v*) of the respective substrate in the same buffer. Activities were expressed as micromole of reducing sugar (glucose or xylose) equivalent released per minute. β-Glucosidase activity was determined by measuring the hydrolysis of *p*-nitrophenyl β-d-glucopyranoside (*p*-NPG) as described previously (Lymar *et al.*, 1995 ▸). The enzyme (1 ml) was incubated with 5 m*M* *p*-NPG in 1 ml 50 m*M* citrate buffer pH 4.5 at 323 K for 60 min, the reaction was stopped by adding 1 ml 1 *M* sodium carbonate and the colour formed was measured at 410 nm. One unit of β-glucosidase activity was defined as the amount of enzyme that liberates 1 µmol *p*-nitrophenol per minute under the assay conditions. Specific activity is defined as the number of units per milligram of protein.

### 2.4. Crystallization   

**Figure 2:**
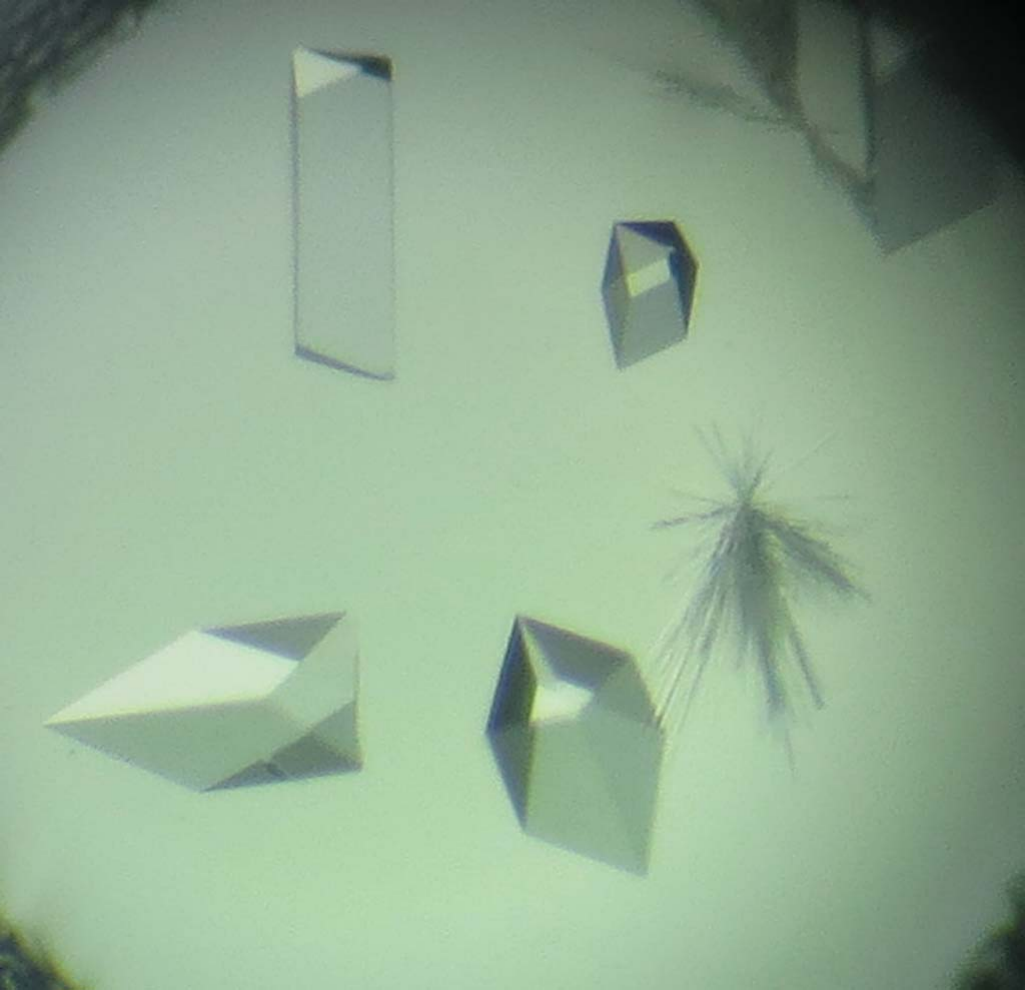
Crystals were obtained in 0.1 *M* glycine–NaOH pH 9.0, 30% PEG 8K, 0.5 *M* potassium chloride by the sitting-drop vapour-diffusion method.

Initial crystallization screening was performed at 293 K by the sitting-drop vapour-diffusion method using commercial crystallization screening kits. Each crystallization drop was prepared by mixing 0.3 µl reservoir solution and 0.3 µl protein solution, and the mixture was equilibrated against 0.1 ml reservoir solution. After four weeks, crystals appeared in a solution consisting of PEG 8K, 0.5 *M* potassium chloride, 0.1 *M* HEPES pH 7.5. Conditions were further optimized by varying the pH value and precipitant concentrations to obtain diffraction-quality crystals (Table 2 ▸). For data collection, the crystals were grown for four weeks at 293 K, with the optimal condition consisting of 0.1 *M* glycine–NaOH pH 9.0, 30% PEG 8K, 0.5 *M* potassium chloride (Fig. 2 ▸).

### 2.5. Data collection, structure determination and refinement   

Prior to data collection, a single crystal was transferred into mother liquor containing 30%(*v*/*v*) glycerol as a cryoprotectant and then mounted in a 0.1 mm nylon loop (Hampton Research) and flash-cooled in liquid nitrogen. X-ray diffraction data were collected to 1.76 Å resolution on beamline BL17U1 at Shanghai Synchrotron Radiation Facility (SSRF; Shanghai, People’s Republic of China) using a charge-coupled device (CCD) detector. The data were processed and scaled using the *HKL*-2000 and *CCP*4 suites (Winn *et al.*, 2011 ▸). Data-collection and processing statistics are shown in Table 3 ▸. The crystal belonged to space group *P*2_1_2_1_2_1_, with unit-cell parameters *a* = 53.57, *b* = 73.26, *c* = 79.20 Å, and contained one molecule in the asymmetric unit. Calculation of the Matthews coefficient using *CCP*4 indicated a *V*_M_ of 2.22 Å^3^ Da^−1^, corresponding to a solvent content of 44.61%. The crystal structure of Cel10 was determined by molecular replacement using the CMCax structure (PDB entry 1wzz, 36% identity; Yasutake *et al.*, 2006 ▸) as the search model in *Phaser* (McCoy *et al.*, 2007 ▸) and was refined with *PHENIX* (Adams *et al.*, 2010 ▸). All molecular figures were prepared using *PyMOL* (Schrödinger). The atomic coordinates and structure factors have been deposited in the Protein Data Bank with accession code 5gy3. Structure-refinement statistics are shown in Table 4 ▸.

## 3. Results and discussion   

### 3.1. Substrate-specificity analysis   

**Figure 3:**
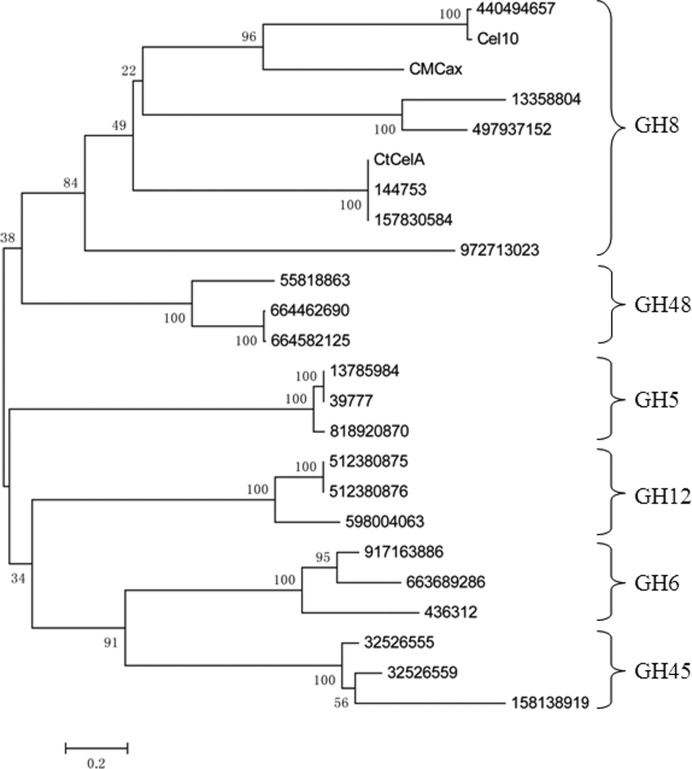
Phylogenetic tree showing the relationship between Cel10 and other hydrolase families. In the phylogenetic tree analysis, Cel10 was in the same cluster as members of different families and showed over 97% homology to Cel8A in GH8 (GenBank accession No. 440494657). These results demonstrate that Cel10 is a member of GH8. The phylogenetic tree was drawn using *MEGA* v.4.0. The amino-acid sequence of Cel10 was aligned with those from other different cellulase hydrolase families to generate a neighbour-joining phylogenetic tree. Bootstrap percentage values are indicated at branch points. Accession numbers are listed in the centre.

The Cel10 enzyme was analyzed using various substrates to determine its catalytic specificity, as shown in Table 5 ▸. The results showed that Cel10 hydrolyzes amorphous CMC and crystalline forms of cellulose (Avicel and xylan) but does not hydrolyze *p*-NPG. However, Cel10 cellulase activity was more efficient on CMC than on Avicel and xylan, which indicates that it is an endoglucanese. Furthermore, Cel10 was considered to be a member of the GH8 family according to the CAZy database (http://www.cazy.org; Cantarel *et al.*, 2009 ▸) and in a phylogenetic analysis with *MEGA*4.0 (Tamura *et al.*, 2007 ▸) from amino-acid sequence comparison of Cel10 with other glycosyl hydrolase family members (Fig. 3 ▸). CMC (an amorphous cellulose derivative) is commonly used as a substrate for the study of endoglucanases (Lynd *et al.*, 2002 ▸). On the other hand, exoglucanases can degrade Avicel efficiently (Lynd *et al.*, 2002 ▸). In our case, Cel10 displayed a stronger catalytic preference for CMC than for Avicel. In a comparison of activity against CMC with other endoglucanases (Schwarz *et al.*, 1986 ▸; Mahadevan *et al.*, 2008 ▸), CtCelA showed the highest enzyme specific activity. Interestingly, the differences between these recombinant endoglucanases illustrate that the enzymatic activity was mainly affected by the original strain specificity (Posta *et al.*, 2004 ▸), the classification of the GH family (Janeček *et al.*, 2011 ▸) and synergism (Lynd *et al.*, 2002 ▸).

Our data suggest that the degree of hydrolysis of an insol­uble substrate might be related to intermolecular synergy between the carbohydrate-binding module (CBM) and the catalytic domain of cellulases, which occurs because binding of the CBM to the cellulose substrate brings the catalytic domain to the substrate surface and the CBM loosens the crystalline structure by partially separating the cellulose strands from the surface of cellulose microfibrils, making the substrate easier to hydrolyze (Lynd *et al.*, 2002 ▸). Therefore, CBM is essential for the hydrolysis of crystalline cellulose (Ogawa *et al.*, 2007 ▸). It has been proposed that these independent ‘domains’ are critical for targeting the enzymes to the substrate and for enhancing their hydrolytic activity. This result suggests that the absence of a CBD in Cel10 makes it less effective against crystalline cellulose.

### 3.2. Three-dimensional structure of Cel10   

**Figure 4:**
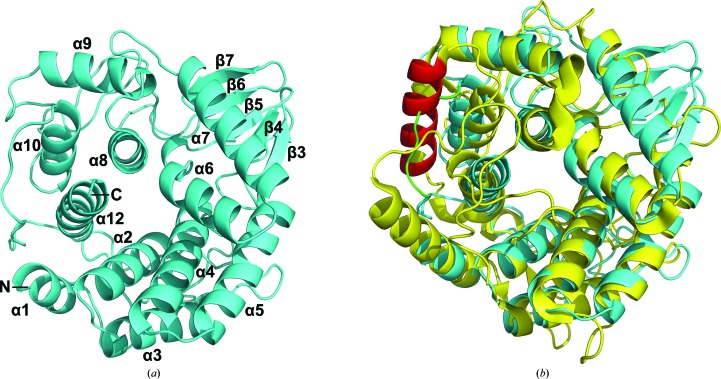
Structure of Cel10 (*a*) and structure superimposition of Cel10 (PDP entry 5gy3; blue) with CtCelA (PDB entry 1kwf; yellow) (*b*). Helix α11 in CtCelA (labelled in red) is missing and forms a flexible loop (labelled in green) in Ce110.

**Figure 5:**
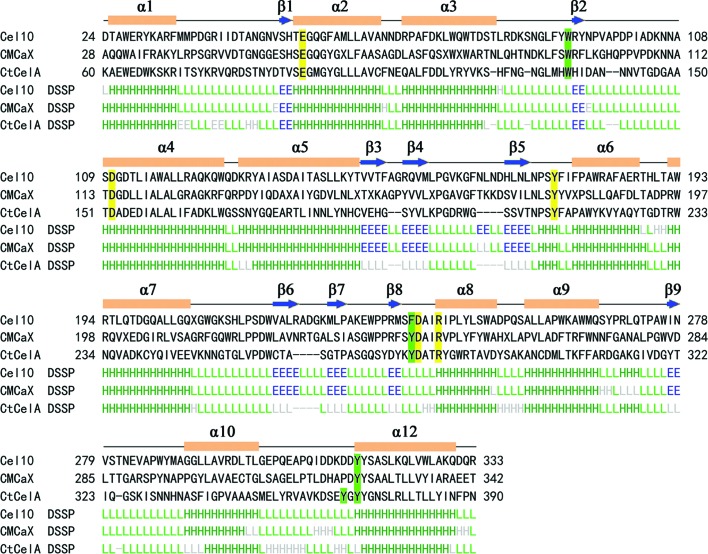
The structure and sequence alignment of Cel10 with CMCax (PDB entry 1wzz) and CtCelA (PDB entry 1kwf). Structure-based sequence alignment of enzymes belonging to GH8. Conserved catalytic residues are highlighted in yellow and the aromatic residues forming sugar-recognition subsites are shown in green. This figure was created using *DaliLite* (Holm & Rosenström, 2010 ▸).

**Figure 6:**
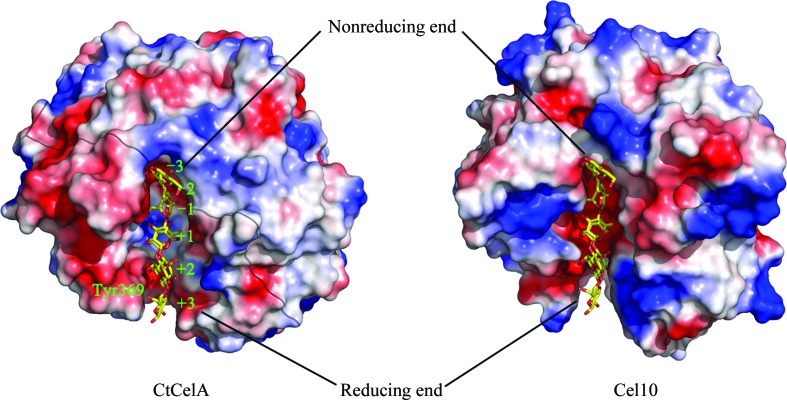
Molecular surface-potential representation of CtCelA (left; PDB entry 1kwf) and Cel10 (right). A model of the substrate in PDB entry 1kwf is also shown in the cleft of Cel10. The electrostatic surface potentials were generated using *PyMOL* (http://www.pymol.org) in absolute mode. Areas coloured white, red and blue denote neutral, negative and positive potential, respectively.

The structure of Cel10 was solved by molecular replacement using the three-dimensional structure of the *Aceto­bacterxylinum* endoglucanase CMCax (PDB entry 1wzz) as the search model. The final structure was refined at 1.76 Å resolution with an *R*_work_ of 16.15% and an *R*_free_ of 19.88% (Table 4 ▸). There is one molecule in the asymmetric unit and the final structure contains residues 24–333. The structure of Cel10 is mainly composed of 11 helices forming an overall so-called ‘barrel fold’ (α1–α12; Fig. 4 ▸ *a*), which differs from most of the other enzymes belonging to the GH8 subfamily, which display an atypical (α/α)_6_-barrel motif fold. It is similar to the CMCax structure but with one helix (α11 in CtCelA, labelled in red) missing in the Ce110 structure; instead a flexible loop is formed (labelled green) (Fig. 4 ▸ *b*). Notably, compared with the flexible loops in the CtCelA structure the connections between helices α5 and α6 and between α7 and α8 form extended β-strands β3, β4 and β5, and β6 and β7, respectively, with two antiparallel β-sheets being formed by β3, β4 and β5 and by β6 and β7 (Fig. 5 ▸). However, the functional role of this stable protrusion, which differs from that in the corresponding part of CtCelA, awaits further investigation. As reported, CtCelA is one of the best-characterized endo-β-1,4-glucan­ases, with the structure having been determined in complex with the cellobiose substrate (Alzari *et al.*, 1996 ▸). According to the phylogenetic analysis, Cel10 should exhibit essentially similar enzymatic characteristics to CtCelA (Fig. 3 ▸). However, structural comparisons of the active sites of Cel10 and CtCelA reveal notable differences. Two of the five aromatic residues involved in stacking interactions that are critical for substrate recognition by CtCelA (Guérin *et al.*, 2002 ▸), corresponding to Trp205 and Tyr369 of CtCelA, are not conserved in Cel10. Phe163 of Cel10 seems to play an identical role to Trp205 of CtCelA, while a residue corresponding to Tyr369 of CtCelA is missing in the Cel10 structure, leading to a significant broadening of the cleft at the cellooligosaccharide reducing end (Fig. 6 ▸). These observations suggested that sugar-recognition subsite −3 is not present in Cel10, implying that Cel10 cannot immobilize cellobiose at the active-site cleft owing to the structural differences in the oligosaccharide recognition site.

Consistent with the homology model of CMCax, *Populus tremula* × *tremuloides* KOR and the expected structure of AgCelC (Master *et al.*, 2004 ▸), the absence of subsite −3 of Cel10 is a common feature among cellulose biosynthesis-related endoglucanases (Yasutake *et al.*, 2006 ▸). It has been speculated that KOR may function in cleavage of the lipid-linked glucose from the reducing end of the growing glucan chain (Peng *et al.*, 2002 ▸), and it has been reported that AgCelC may act as a transferase rather than as an endoglucanase during cellulose synthesis (Matthysse *et al.*, 1995 ▸). The absence of subsite −3 may account for the recognition of such lipid-linked oligosaccharides. However, the relationship between cellulose synthesis and lipid-linked oligosaccharides in *A. xylinum* has not yet been clarified, and the actual role of Cel10 in the cellulose-production process requires further investigation.

## Supplementary Material

PDB reference: Cel10, 5gy3

Chemical structures of the substrates.. DOI: 10.1107/S2053230X16017891/hv5341sup1.pdf

## Figures and Tables

**Table 1:** Macromolecule-production information

Source organism	*K. pneumoniae*
DNA source	cDNA from *K. pneumoniae*
Forward primer	CGGGATCCGATACGGCCTGGGAGCGCTA
Reverse primer	CCGCTCGAGCTAACGCTGATCCTGTTTCG
Cloning vector	pET-32a
Expression vector	pET-32a
Expression host	*E. coli* BL21 (DE3)
Complete amino-acid sequence of the construct product	GTSSMADIGSDTAWERYKARFMMPDGRIIDTANGNVSHTEGQGFAMLLAVANNDRPAFDKLWQWTDSTLRDKSNGLFYWRYNPVAPDPIADKNNASDGDTLIAWALLRAQKQWQDKRYAIASDAITASLLKYTVVTFAGRQVMLPGVKGFNLNDHLNLNPSYFIFPAWRAFAERTHLTAWRTLQTDGQALLGQMGWGKSHLPSDWVALRADGKMLPAKEWPPRMSFDAIRIPLYLSWADPQSALLAPWKAWMQSYPRLQTPAWINVSTNEVAPWYMAGGLLAVRDLTLGEPQEAPQIDDKDDYYSASLKQLVWLAKQDQR

**Table 2:** Crystallization

Method	Sitting drop
Plate type	Cryschem plate
Temperature (K)	293
Protein concentration (mg ml^−1^)	28
Buffer composition of protein solution	20 m*M* Tris–HCl pH 6.0, 150 m*M* NaCl, 5% glycerol
Composition of reservoir solution	0.1 *M* glycine–NaOH pH 9.0, 30% PEG 8K, 0.5 *M* KCl
Volume and ratio of drop	2 µl (1:1 ratio)
Volume of reservoir (µl)	400

**Table 3:** Data collection and processing Values in parentheses are for the outer shell.

Diffraction source	BL17U1, SSRF
Wavelength (Å)	0.9792
Temperature (K)	100
Detector	ADSC Q315R
Crystal-to-detector distance (mm)	250
Rotation range per image (°)	1
Total rotation range (°)	180
Exposure time per image (s)	0.8
Space group	*P*2_1_2_1_2_1_
*a*, *b*, *c* (Å)	53.570, 73.256, 79.200
α, β, γ (°)	90, 90, 90
Mosaicity (°)	0.3
Resolution range (Å)	50–1.7639
Total No. of reflections	130639
No. of unique reflections	30441 (1508)
Completeness (%)	99.23
Multiplicity	4.3 (4.4)
Wilson *B* factor (Å^2^)	14.48
〈*I*/σ(*I*)〉	32.98 (10.02)
CC_1/2_	0.954
*R*_merge_ †	0.08 (0.236)
*R*_r.i.m._ ‡	0.043 (0.108)

† *R*_merge_ = 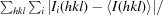 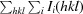, where *I_i_*(*hkl*) are the intensities of the individual replicates of a given reflection *hkl* and 〈*I*(*hkl*)〉 is the average intensity over all replicates of that reflection.

‡ Estimated *R*_r.i.m_ = *R*_merge_[*N*/(*N* – 1)]^1/2^, where *N* is the data multiplicity.

**Table 4:** Structure determination and refinement

Resolution range (Å)	44.361–1.763
Completeness (%)	99.55
No. of reflections, working set	31307
No. of reflections, test set	1517
Final *R*_work_	0.1615
Final *R*_free_	0.1988
No. of non-H atoms	
Total	2495
Water	480
Total	2975
R.m.s. deviations	
Bonds (Å)	0.006
Angles (°)	0.840
Average *B* factor (Å^2^)	17.0
Ramachandran plot	
Favoured regions (%)	97.08
Additionally allowed (%)	2.92
Outliers (%)	0
PDB code	5gy3

**Table 5:** Activity of endoglucanase Cel10 towards various substrates

Substrate	Activity (U mg^−1^)
CMC	31.8
Avicel	18.3
Xylan	8.7
*p*-NPG	None
