# Supplementary material for: The crystal structure of the endoglucanase Cel10, a family 8 glycosyl hydrolase from Klebsiella pneumoniae
Source: Acta Crystallogr F Struct Biol Commun. 2016 Nov 25;72(Pt 12):870–6. doi: 10.1107/S2053230X16017891 (PMC5137463; doi:10.1107/S2053230X16017891)
Supplement: Supplementary file 1 [file f-72-00870-sup1.pdf]

Acta Cryst F STRUCTURAL BIOLOGY COMMUNICATIONS

Volume 72 (2016)

Supporting information for article:

The crystal structure of the endoglucanase Cel10, a family 8 glycosyl hydrolase from *Klebsiella pneumoniae*

Ayman Attigani, Lifang Sun, Qing Wang, Yadan Liu, Dingping Bai, Shengping Li and Xiaohong Huang

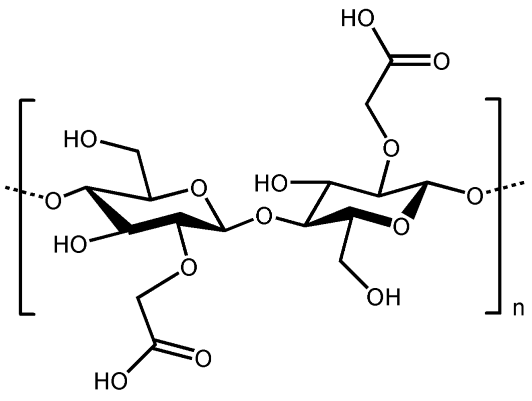

CMC

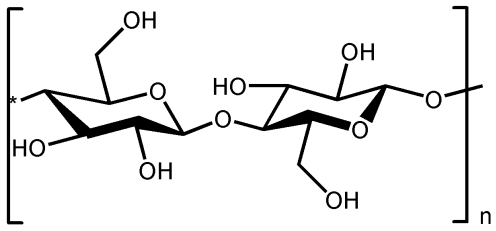

Avicel

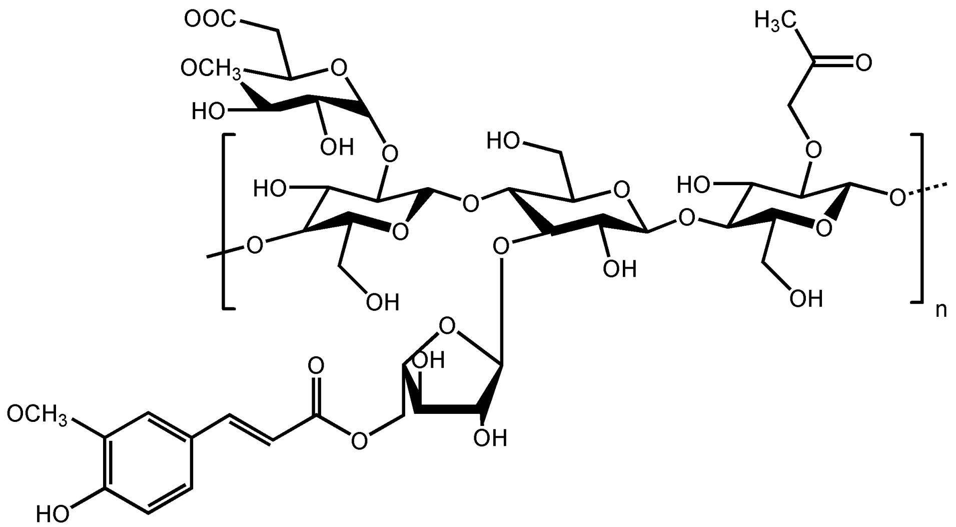

Xylan

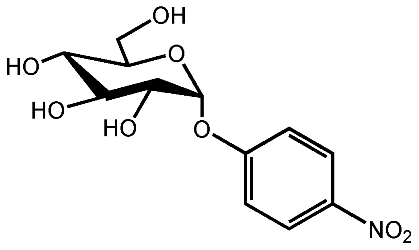

P-NPG

Figure S1 Chemical structures of the substrates.
